# The relationship between triglyceride levels and medication overuse headache in patients with chronic migraine

**DOI:** 10.22514/jofph.2026.027

**Published:** 2026-03-12

**Authors:** Hailin Liu, Hefei Tang, Zhihui Duan

**Affiliations:** ^1^Department of Neurology, Luoyang Central Hospital Affiliated to Zhengzhou University, 471000 Luoyang, Henan, China; ^2^Beijing Tiantan Hospital, Capital Medical University, 100070 Beijing, China; ^3^National Clinical Research Center for Neurological Disease, 100070 Beijing, China

**Keywords:** Medication overuse headache, Chronic migraine, Dyslipidemia

## Abstract

**Background**: Emerging evidence suggests that disturbances in lipid metabolism play a significant role in the etiology of migraines. However, the relationship between lipid metabolism disturbances and medication overuse headache (MOH) remains uncertain. This study aims to evaluate the association between elevated triglyceride (TG) levels and the occurrence of MOH among patients with chronic migraine (CM). **Methods**: A total of 267 hospitalized individuals diagnosed with CM were enrolled into the study. The participants were divided into two distinct groups based on the presence or absence of MOH. Questionnaires were employed for gathering demographic data, and a systematic inquiry was conducted to ascertain the overall prevalence of headaches. The laboratory examination, anxiety and depression scale, Pittsburgh sleep quality index, and other components were used to evaluate improvement. Initially, the study data underwent univariate analysis, whereby indicators demonstrating statistical significance (*p* < 0.05) were chosen as independent variables. Subsequently, a binary logistic regression analysis was conducted. **Results**: The cohort included a total of 87 patients without MOH and 180 patients with MOH. The following five risk variables emerged via single-factor analysis (*p* < 0.05): headache duration (years), headache frequency (days/months), headache intensity measured by a visual analogue scale (0–10), diabetes history, and TG levels. Elevated TG was associated with greater odds (OR = 1.498 (1.018, 2.259), *p* = 0.037) of MOH after accounting for demographics, headache features, and cardiometabolic comorbidities (including diabetes and hypertension). **Conclusions**: Elevated TG levels may be associated with MOH in patients with CM, underscoring their importance in the diagnostic evaluation and therapeutic management of patients with CM.

## 1. Introduction

Migraine is a debilitating neurological condition characterized by recurrent episodes of headaches accompanied by various neurological and systemic manifestations. Repeated assaults over a long period may have devastating effects on an individual’s health, quality of life, and ability to contribute to society. It has gained recognition as an escalating global health burden. Epidemiological evidence shows that migraines occur in roughly 10% of men and 22% of women over the course of a lifetime [1]. Chronic migraine (CM) is defined as headache occurring on more than 15 days per month, for at least three months [2, 3].

Medication overuse headache (MOH) is a condition in which people with CM consume excessive doses of pain medication, exacerbating their CM symptoms [4]. Primary headaches, particularly migraines, are the only headache disorders associated with MOH [5, 6]. According to the International Classification of Headache Disorders, Third Revision (ICHD-3), it is permissible to diagnose MOH and CM concurrently [2]. Worldwide, the incidence of MOH is estimated between 0.7% and 1%. MOH is a global socioeconomic burden related to significant long-term illness, impairment, and reduced quality of life, particularly in less developed countries [7]. The societal cost of MOH treatment is approximately threefold than that of headache treatment for individuals [8, 9]. Migraine is linked to MOH and is the second biggest cause of disability, according to the Global Burden of Disease 2016 [1].

Recent findings suggest that blood lipid levels may play a role in migraine frequency and intensity [10, 11, 12, 13]. Low-and high-density lipoprotein cholesterol (LDL- and HDL-C), and total cholesterol (TC) were all shown to be higher in individuals with migraine in a large population-based investigation [14]. An association has been identified between triglycerides (TG) levels [15], a common genetic component [16] and migraine. Emerging evidence suggests that dyslipidemia and lipid-related markers correlate a greater migraine burden and chronification risk possibility through lipid mediators and Free fatty acids (FFAs). MOH itself is a strong risk factor for migraine chronification [7, 17, 18, 19, 20, 21, 22], potentially raising questions whether there is a connection between lipid metabolism disorder and MOH. Beyond migraine, disordered lipid metabolism has been implicated in multiple pain conditions. In CM, targeted lipidomic profiling of plasma and cerebrospinal fluid has revealed altered fatty-acid composition, desaturase/elongase indices, and signaling lipids, consistent with abnormal lipid handling and impaired energy homeostasis [23]. At the population level, dysregulated lipid pathways and obesity-related lipid indices have also been linked with severe headache or migraine [10]. In temporomandibular disorders, erythrocyte polyunsaturated fatty acids (PUFAs) and higher omega-6:omega-3 ratios have been linked with greater pain and symptom burden, thereby supporting a link between PUFA balance and nociplastic orofacial pain [24]. Similarly, in fibromyalgia, metabolomic and lipidomic analyses have reported alterations in lipid classes, such as lysophosphatidylcholines, lysophosphatidylethanolamines, and TG, compared with controls, suggesting broader perturbations in lipid metabolism in centralized pain states [25]. Collectively, these findings provide the rationale for examining circulating TG and their potential association with MOH in CM.

Accordingly, this study aimed to evaluate differences in blood lipid profiles between CM patients with and without MOH, and to investigate potential associations between lipid metabolic disturbances and MOH.

## 2. Method

### 2.1 Study procedures and data collection

Ethical approval for this study was approved from the Institutional Review Board of Beijing Tiantan Hospital, Capital Medical University. Eligibility included patients admitted to Beijing Tiantan Hospital, affiliated to Capital Medical University, and Luoyang Central Hospital, affiliated to Zhengzhou University, between July 2022 and May 2025 with a new diagnosis of CM (with or without MOH). Prior to enrollment, written informed consent was obtained from all participants. Demographic data, including age and sex, and contact information for clinical follow-up were recorded. Additionally, history of headache was collected using structured interviews. To ensure confidentiality, personal identifiers were securely stored and excluded from all analyses. Fasting venous blood samples were obtained from all participants for the lipid panel assessment and routine laboratory testing. Additional tests, including electrocardiography, vascular ultrasound (such as transcranial Doppler, carotid duplex) when clinically indicated, echocardiography, abdominal ultrasound, and advanced neuroimaging (brain magnetic resonance imaging (MRI)/functional magnetic resonance imaging (fMRI) or positron emission tomography (PET)/magnetic resonance (MR)), were performed in subsets at the discretion of the attending neurologist. Imaging results were not used to define exposures or outcomes, nor were they included as covariates in the regression models.

### 2.2 Inclusion and exclusion criteria

A within-CM comparative design was employed to evaluate associations between lipid measures and MOH among patients with CM, excluding individuals without CM. At the initial appointment, patients underwent a comprehensive consultation with an expert in headache medicine, a physical examination and a questionnaire assessment. Two or more neurologists independently diagnosed all migraine patients using the diagnostic criteria established by the ICHD-3 [2]. Briefly, MOH was characterized as headache occurrence for ≥15 days per month in a patient with a pre-existing headache disorder alongside regular overuse of acute/symptomatic headache medication for >3 months. Overuse thresholds were established in accordance with the ICHD-3 subtype criteria as follows: ergotamines, triptans, opioids, or combination-analgesic medications on ≥10 days/month; simple analgesics (paracetamol/acetylsalicylic acid/other nonsteroidal anti-inflammatory drugs (NSAIDs)) on ≥15 days/month; or any combination of acute medications on ≥10 days/month. Classification of cases was determined via structured interviews that documented, for each acute medication, the type, dose, and number of days used per month over the preceding 3 months. Participants who reported medication-associated headaches with overuse lasting less than 3 months were classified as having acute medication overuse (AMO) and subsequently were not categorized as MOH. A neurologist specializing in headache medicine confirmed the diagnosis. Patients who (a) met the inclusion criteria and (b) were between the ages of 18 and 65 years, and (c) met the ICHD-3 criteria for CM were included.

The exclusion criteria was based on the following: (i) presence of other chronic pain conditions (such as fibromyalgia); (ii) additional primary headache disorders (including cluster headache); (iii) presence of facial neuralgia/neuropathy; (iv) presence of secondary headache; (v) diagnosis with acute headache with symptom duration <1 month; (vi) incomplete key clinical or laboratory data; and (vii) current use (within the preceding 3 months) of lipid-modifying medications, including statins, fibrates, ezetimibe, omega-3 fatty-acid formulations, niacin, or proprotein convertase subtilisin/kexin type 9 (PSCK9) inhibitors.

Although participants with cardiometabolic comorbidities, such as diabetes and hypertension, were included, these factors served as covariates in the multivariable analyses.

### 2.3 Participants’ assessments

The questionnaire was designed to gather demographic and clinical data of headache patients, including age, gender, race/ethnicity, education level, employment status, drug usage, and prevalence of mental health conditions. The Hamilton Depression Rating Scale (HAMD) and the Hamilton Anxiety Scale (HAMA) were, respectively, utilized to assess symptoms of depression and anxiety. The Pittsburgh Sleep Quality Index (PSQI) was employed to evaluate the occurrence and severity of sleep disruptions over the course of the previous month. Participants were subjected to overnight fasting (≥12 hours) before venous blood sample collection. The lipid panel assessments encompassed total cholesterol (TC), TG, high-density lipoprotein cholesterol (HDL-C), low-density lipoprotein cholesterol (LDL-C), apolipoprotein A-I (APO-A1), and apolipoprotein B (APO-B). Notably, non-high-density lipoprotein cholesterol (non-HDL-C) was computed as: non-HDL-C = TC − HDL-C. Headache intensity was measured using a visual analogue scale (VAS, 0–10) at the enrolment visit (0 = “no pain”; 10 = “worst imaginable pain”). Participants reported their average over the past 30 days headache intensity. Headache frequency was defined as the total number of headache days per month, irrespective of whether the pain was attributed to migraine attacks or to medication overuse. At baseline, frequency data was collected using structured interviews, reflecting the patients’ recall of the previous 3 months. Headache medication use, both acute and preventive, during this period was also determined. For acute medications, information was recorded on drug class, specific agent, dose, and monthly frequency of use. For patients classified as MOH, the classes of overused acute medications are summarized in **Supplementary Table 1**.

### 2.4 Handling of missing data

Participants were required to provide complete baseline questionnaire and scale data, including the structured headache interview and validated instruments, such as VAS, HAMA, HAMD, and PSQI. Incomplete questionnaires and scales (missing responses precluding CM/MOH classification or key covariates) were excluded from the analysis; notably, logistic regression analyses were performed on a complete-case dataset. For all variables included in the models, no missing values were present; excluding the need for imputation.

### 2.5 Statistical analysis

Continuous variables were reported as mean ± standard deviation (SD) or median (interquartile range, IQR), as appropriate, while categorical variables were presented as frequencies, n (percentages, %). Normality was assessed using the Shapiro-Wilk test. Between-group comparisons (specifically, CM with MOH *vs.* CM without MOH) used the two-sample *t* test or Wilcoxon rank-sum test for continuous variables and the χ^2^ test (or Fisher’s exact test when expected counts were <5) for categorical variables. Logistic regression was performed with MOH status (yes/no) as the dependent variable. Serum TG concentration (mmol/L) was the primary exposure and was modeled as a continuous variable. Covariates were specified a priori. Three prespecified models were fit: (i) Crude—unadjusted association of TG with MOH (no covariates); (ii) Model 1—adjusted for clinical headache phenotype/severity: disease duration (years), total headache days/month, VAS pain score, and diabetes (yes/no) as a core metabolic confounder; (iii) Model 2—further adjusted for broader cardiometabolic confounders: age (years), body mass index (BMI) (kg/m^2^), hypertension (yes/no), with diabetes retained to ensure consistent control of this key confounder across models. Logistic regression coefficients (β) were exponentiated and presented as odds ratios (OR = exp(β)) with 95% confidence intervals (CI) for interpretability. All analyses were performed using SPSS 26 (IBM Corp., Armonk, NY, USA), with *p*-value < 0.05 indicating statistical significance.

## 3. Results

At the time of first hospitalization, 355 patients with CM, including 273 females, with a mean age of 47.0 (33.0–54.0) years, were screened. Of these participants, 38 were eliminated owing to being too old, and another 50 were disqualified due to incomplete scale data. Ultimately, 267 CM patients were included (200 females), with a mean age of 47.0 (36.0–55.0) (Fig. 1). Of these participants, 180 had MOH, including 143 females, with a mean age of 46 (37.0–52.0), and 87 did not, including 57 females, with a mean age of 48.0 (31.0–54.0) (Table 1).

**Fig. 1.** Patient enrollment process. CM: chronic migraine; MOH: medication overuse headache.

**Table 1.** Baseline characteristics of the included study participants.

		CM with MOH (n = 180)	CM Without MOH (n = 87)	*p* value
Age, yr		46 (37.0, 52.0)	48.0 (31.0, 54.0)	0.057
Age of onset (yr)		22.0 (14.0, 31.0)	23.0 (18.0, 35.0)	0.351
Female, n (%)		143 (79.4%)	57 (65.5%)	0.423
BMI category, n (%)				
	<18.5	13 (7.2%)	8 (9.2%)	0.070
	18.5–24.9	102 (56.7%)	48 (55.2%)	
	>24.9 and <30	51 (28.3%)	27 (31.0%)	
	≥30	14 (7.8%)	4 (4.6%)	
Duration of disease (yr)		21.0 (14.0, 31.0)	15.1 (6.0, 24.0)	<0.001
Headache frequency (d/mon)		31.0 (15.7, 31.1)	21.0 (15.5, 31.4)	0.040
VAS pain score (0–10)		7.4 (6.4, 7.9)	3.9 (5.4, 8.2)	0.020
Headache Family history, n (%)		50 (27.8%)	25 (28.7%)	0.792
History of hypertension, n (%)		25 (13.9%)	16 (18.4%)	0.295
Diabetes, n (%)		4 (2.2%)	11 (12.6%)	0.015
HCY, μmol/L		11.20 (8.33, 12.98)	9.74 (7.99,10.87)	0.249
TG, mmol/L		1.38 (0.99, 1.79)	0.97 (0.71, 1.62)	0.001
TC, mmol/L		4.63 (4.15, 5.32)	4.35 (3.79, 5.32)	0.398
HDL-C, mmol/L		1.38 (1.21, 1.57)	1.34 (1.24, 1.70)	0.601
LDL-C, mmol/L		2.68 (2.19, 3.18)	2.46 (2.17, 3.70)	0.587
APO-A1, g/L		1.29 (1.17, 1.61)	1.26 (1.11, 1.50)	0.151
APO-B, g/L		0.79 (0.68, 0.91)	0.75 (0.64, 0.89)	0.444
Non-HDL-C, mmol/L		3.21 (2.82, 3.81)	2.87 (2.35, 3.91)	0.198
Educational level, n (%)				
	College or above	76 (42.3%)	43 (49.4%)	0.301
	High school or technical secondary school	36 (20.0%)	18 (20.7%)	
	Junior high school	35 (19.4%)	14 (16.1%)	
	Primary school	24 (13.3%)	7 (8.0%)	
	Illiteracy	9 (5%)	5 (5.8%)	
Anxiety scale (points)		7 (5.0, 9.0)	8 (4.0, 13.0)	0.557
Depression scale (points)		6 (4.0, 8.0)	5 (2.0, 11.0)	0.424
PSQI (points)		(10.12 ± 5.01)	(9.35 ± 5.11)	0.887

*Data are expressed as mean ± SD or median (IQR) for continuous variables, and n (%) for categorical variables. Headache frequency reflects total headache days per month. Non-HDL-C was calculated as TC − HDL-C. CM: chronic migraine; MOH: medication overuse headache; BMI: body mass index; VAS: visual analogue scale; HCY: homocysteine; TG: triglycerides; TC: total cholesterol; HDL-C: high-density lipoprotein cholesterol; LDL-C: low-density lipoprotein cholesterol; APO-A1: apolipoprotein AI; APO-B: apolipoprotein B; Non-HDL-C: non-high-density lipoprotein cholesterol; PSQI: Pittsburgh Sleep Quality Index.*

### 3.1 Comparing CM patients with and without MOH

Compared with patients without MOH, those with MOH exhibited a longer duration of headache (21.0 (14.0–31.0) *vs.* 15.1 (6.0–24.0), *p * < 0.001), a higher frequency of headache (31.0 (15.7–31.1) *vs.* 21.0 (15.5–31.4), *p* = 0.040), a higher VAS score (7.4 (6.4–7.9) *vs.* 3.9 (5.4–8.2), *p* = 0.020), and a smaller proportion of individuals with a history of diabetes (2.2% *vs.* 12.6%, *p* = 0.015). However, there were no significant differences observed in hypertension, BMI, education level, gender, family history of headache, PSQI, anxiety, and depression between the two groups (Table 1).

### 3.2 Elevated TG levels are a risk factor for MOH

Patients with MOH had greater TG values (1.38 (0.99, 1.79) *vs.* 0.97 (0.71, 1.62), *p* = 0.001) than those without MOH. An increased risk of MOH was observed in CM patients who had high TG levels (OR = 1.498 (1.018, 2.259), *p* = 0.037), after controlling for disease duration, VAS, headache frequency, and diabetes, (OR = 1.755 (1.084, 2.723), *p* = 0.026), and after accounting for diabetes, age, BMI, and hypertension (OR = 1.621 (1.023, 2.584), *p* = 0.039) (Table 2).

**Table 2.** Association between fasting TG and MOH in chronic migraine.

	OR (95% CI) per 1 mmol/L higher TG	*p*-value
Crude	1.498 (1.018, 2.259)	0.037
Model 1	1.755 (1.084, 2.723)	0.026
Model 2	1.621 (1.023, 2.584)	0.039

*Outcome = MOH (yes/no). Exposure = fasting triglycerides (TG, mmol/L; continuous), effect per 1 mmol/L increase. Crude model: unadjusted (no covariates). Model 1: adjusted for disease duration (years), total headache days/month, VAS pain score, and diabetes (yes/no). Model 2: adjusted for diabetes (yes/no), age (years), BMI (kg/m^2^), and hypertension (yes/no). ORs are from logistic regression. OR: odds ratio; CI: confidence interval.*

## 4. Discussion

Investigating the natural progression of MOH, including identifying risk factors for its onset, is crucial for guiding the selection or development of effective therapies. Our research demonstrated that TG levels were higher in CM patients with MOH compared with those without MOH. However, there were no significant differences between the groups in terms of TC, HDL, LDL, apolipoprotein A-I (APO-A1), apolipoprotein B (APO-B), or non-high-density lipoprotein cholesterol (non-HDL-L). After adjusting for potential confounding factors, including disease duration, VAS, headache frequency, diabetes, age, BMI, and hypertension, logistic regression revealed that elevated TG levels were associated with an increased risk of MOH. These findings suggest that hypertriglyceridemia may represent a key comorbidity in MOH, supporting the potential value of incorporating lipid profiling into baseline assessment and holistic management strategies, although the interventional implications remain to be determined.

In this inpatient cohort, higher fasting TG levels were associated with MOH among patients with CM. This within-CM comparison reduces heterogeneity arising from differing primary headache diagnoses but does not allow for causal inference. Notably, CM has been linked to disturbances in energy homeostasis, nociceptive pathways, and inflammatory signals, providing biological plausibility for exploring lipid metabolism in this context [23]. From a clinical prospective, these findings endorse the integration of lipid profiling, including TG, into baseline assessments of MOH patients. Moreover, several comorbid disorders are associated with MOH. Previous studies have shown that patients with MOH have an increased risk of developing chronic musculoskeletal illnesses, insomnia, hypothyroidism, gastrointestinal issues, metabolic syndrome, hypertension, and smoking compared with the general population [26, 27, 28, 29, 30]. Importantly, distinguishing hypertriglyceridemia as a comorbidity from a risk factor for MOH may be challenging in people with both conditions. It is possible that those with a metabolic predisposition to MOH may have their condition triggered or exacerbated by abnormally high levels of TG. Overuse of pain medications, mental instability, disturbed sleep, and reduced physical activity are all comorbidities of MOH that might contribute to hypertriglyceridemia. However, previous studies involving women outpatient data linked MOH occurrence to abdominal obesity and hypertension but not TG [29]. Our inpatient cohort study included both sexes and likely reflects a different case-mix and severity spectrum. Additionally, our analysis modeled fasting TG as a continuous exposure rather than focusing on metabolic-syndrome constructs. These design and analytic differences may account for the discrepancies with previous findings; thus, our findings should be interpreted as associative pending prospective confirmation of causality.

Potential biological links between dyslipidemia and headache burden should be regarded as hypothesis-generating and warrant further investigation. A dyslipidemic, pro-inflammatory milieu, mediated by adipokines and cytokines derived from adipose tissue, may lower nociceptive thresholds and facilitate central sensitization in migraines [31, 32]. Endothelial dysfunction, characterized by impaired nitric oxide bioavailability, could modulate trigeminovascular reactivity and vascular tone, key factors for migraine pathophysiology [33]. Alterations in lipid signaling and free-fatty-acid flux have been observed in CM and may influence neuronal energy sensing and neuropeptide release [23]. Conversely, behavioral and treatment-related factors common in MOH, such as reduced physical activity, poor sleep, and frequent analgesic exposure, may secondarily alter lipid profiles, raising the possibility of reverse causation [34]. The pathophysiology of MOH is complex, likely arising due to a complex interplay of multiple genetic, environmental, and biopsychosocial variables. The onset, etiology, and progression of MOH may be influenced by a combination of genetic vulnerability and diverse biological, behavioral, psychological, and environmental factors [35, 36]. Further research is warranted to validate our findings and elucidate the underlying mechanisms. Although the present research reveals a relationship between TG and MOH, the directionality of this relationship should be clarified by longitudinal studies following MOH patients at baseline with normal TG levels.

Standardized data collection was ensured by the inpatient setting, with data collection comprising overnight fasting blood sampling for the lipid panel, structured interviews, and neurologist-confirmed classification of CM and MOH using the ICHD-3. Headache specialists collected patient clinical information, performed neurological examinations, and administered validated scales according to standardized ward procedures. These procedures likely enhanced measurement consistency and data completeness. This study employed reliable neuropsychological measures. By comprehensively assessing patient lipid profile and reviewing medical history related to conditions affecting lipid metabolism, we identified potential confounding factors and accounted for them in our analysis.

Although this study offers valuable insights, several limitations should be acknowledged. While our findings suggest a potential association between elevated TG and MOH among patients with CM, the cross-sectional, hospital-based design precludes causal inference. Residual confounding and the possibility of reverse causation, whereby MOH-related behavioral changes influence TG, cannot be excluded. Prospective cohort studies and interventional trials are needed to determine whether modifying TG affects MOH risk. In addition to limiting causal inference, the study design may also restrict generalizability beyond inpatients studied at the two tertiary centers in North and Central China. Furthermore, it should be noted that the headache frequency reflects total headache days per month, with no distinction between primary migraine attacks and secondary headaches provoked by medication overuse. This may partially account for the higher frequency observed in the MOH group, and thus the findings must be interpreted accordingly. In addition, residual confounding cannot be excluded. Current users of lipid-lowering medications were excluded at screening, detailed medication data (drug class, dose, duration, timing of last use) were not collected in case report forms for the enrolled cohort; therefore, we could not analyze dose-response or prior (historic) exposure, and residual confounding from past use cannot be excluded. Finally, the study did not include an external control group without CM and MOH. Although the within-CM design reduces heterogeneity and targets a clinically relevant comparison (CM + MOH *vs.* CM without MOH), it limits generalizability and precludes evaluation of whether lipid abnormalities differ between CM and non-CM populations.

## 5. Conclusions

In this cohort of CM patients, elevated TG levels were associated with the presence of MOH, highlighting the importance of considering underlying dyslipidemia in clinical management. Notably, these findings should be viewed as hypothesis-generating, with prospective studies or randomized interventions required to validate this relationship and elucidate the underlying mechanisms.

## Supplementary Material

Supplementary material associated with this article can be found, in the online version, at https://files.jofph.com/ files/article/2031986728569323520/attachment/ Supplementary%20material.docx.


