# Supplementary material for: The relationship between triglyceride levels and medication overuse headache in patients with chronic migraine
Source: J Oral Facial Pain Headache. 2026 Mar 12;40(2):120–6. doi: 10.22514/jofph.2026.027 (PMC13036612; doi:10.22514/jofph.2026.027)
Supplement: Supplementary file 1 [file Supplementary-material.docx]

Supplementary material

Supplementary Table 1. Types of acute analgesics overused by patients with chronic migraine (CM) and medication overuse headache (MOH).

| Acute analgesic type | n (%) |
| --- | --- |
| Compound analgesics | 88 (48.92) |
| Compound cold preparations | 25 (13.67) |
| Non-steroidal anti-inflammatory drugs (NSAIDs) | 33 (18.35) |
| Opioids | 8 (4.68) |
| Caffeine-containing agents | 2 (1.07) |
| Ergotamine tablets | 1 (0.36) |
| Paracetamol (acetaminophen) | 7 (3.96) |
| Triptans | 16 (8.99) |
| **Total** | **180 (100)** |
